# Supplementary material for: Effect of parental adverse childhood experiences on intergenerational DNA methylation signatures from peripheral blood mononuclear cells and buccal mucosa
Source: Transl Psychiatry. 2024 Feb 12;14:89. doi: 10.1038/s41398-024-02747-9 (PMC10859367; doi:10.1038/s41398-024-02747-9)
Supplement: Supplementary file 1 — Supplemental [file 41398_2024_2747_MOESM1_ESM.docx]

**Supplementary Tables and Figures**

***Supplementary Figure 1: Gestational Age at Sample Collection (n = 120, N = 240)***


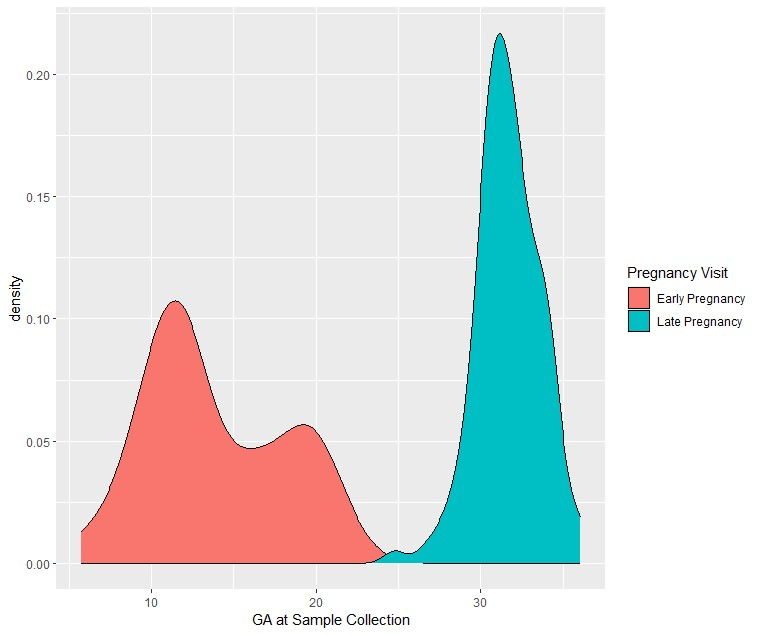


*Gestational Age at sample collection in weeks on the x-axis and probability density function on the y-axis. Individuals were categorized by their early or late pregnancy visit.*

***Supplementary Figure 2: DNA methylation Quality Control in Analytic Population***


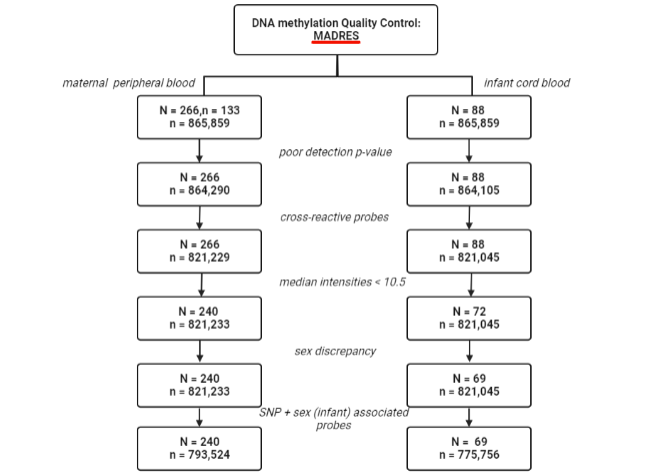


*Poor detection p-value consists of those probes who have no statistically significant (p<0.01) detection signals compared to control probes using the detectionP() function in minfi [1]. Cross-reactive probes are those probes who map to multiple genes. Individuals with a median intensity of probes < 10.5 were removed [1]. Individuals with a sex discrepancy based on reported and predicted sex in minfi were dropped. Finally, SNP-associated probes and sex-associated probes (restricted to neonates) were removed from the analytic dataset [1].* *For the mediation analysis, N = 60 maternal-child paired data was used given the limit of participants in cord blood and limitation of overlapping paired maternal blood samples.*

***Supplementary Figure 3: DNA methylation Quality Control in Replication Population***

**
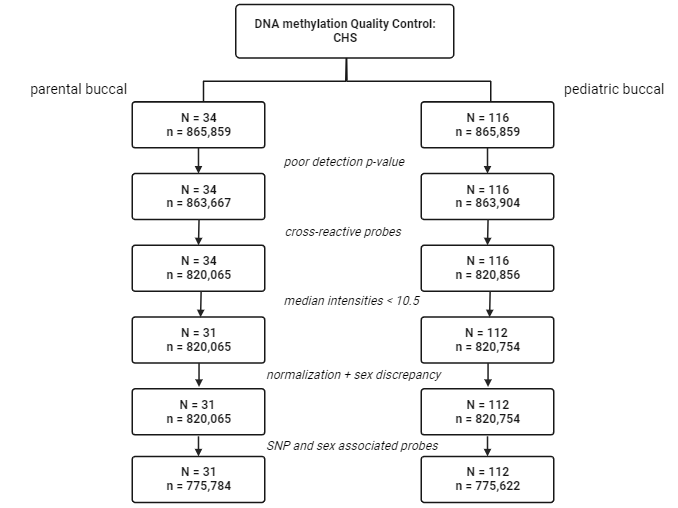
**

*Epithelial cell fractions were generated using HePiDISH [2] and restricted to participants with a fraction greater than 75%. All other quality control indices applied in the discovery cohort were also applied in the replication population.*

***Supplementary Figure 4: CDC-Kaiser Adverse Childhood Experiences Questionnaire***


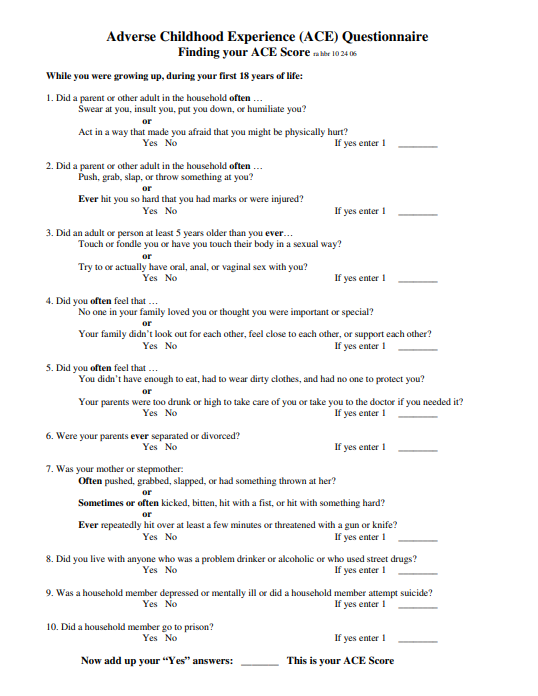


*The CDC-Kaiser based ACE questionnaire [3].*

***Supplementary Table 1: Baseline Characteristics of Parental Replication Sample (N = 30)***

| ACE categories |  | No ACE ACE 1-3 | | ACE 4-6 | | ACE > 6 | | P-Value^A^ | | |
| --- | --- | --- | --- | --- | --- | --- | --- | --- | --- | --- |
|  | 15(48%) | | 10(35%) | | 4(13%) | 1(3.2%) |  | |  |  |
| **Age at Sample Collection** |  | |  | |  |  |  | |  |  |
| Age in Years(SD) | 33.6(4.0) | | 34.5(1.6) | | 34.8(1.6) | 31.3(0) | 0.71 | |  |  |
| **Sex N(%)** |  | |  | |  |  |  | |  |  |
| Female? | 8(53) | | 5(50) | | 3(75) | 1(100) | 0.71 | |  |  |
| **Smoking Status N(%)** |  | |  | |  |  |  | |  |  |
| Ever Smoked? | 4(27) | | 3(30) | | 2(50) | 0(0) |  | |  |  |
| **Hispanic Ethnicity N(%)** |  | |  | |  |  | 0.65 | |  |  |
| Yes | 3(20) | | 3(27) | | 0(0) | 0(0) |  | |  |  |
| **Depression N(%)** |  | |  | |  |  |  | |  |  |
| Ever diagnosed by a doctor? | 2(13) | | 3(30) | | 1(25) | 1(100) | 0.19 | |  |  |
| **Anxiety N(%)** |  | |  | |  |  |  | |  |  |
| Ever diagnosed by a doctor? | 3(25) | | 4(36) | | 2(50) | 1(100) | 0.28 | |  |  |
| **Collection Method N(%)** |  | |  | |  |  |  | |  |  |
| Toothbrush | 4(26) | | 6(60) | | 3(75) | 0(0) | 0.28 | |  |  |
| Swab | 11(73) | | 4(40) | | 1(25) | 1(100) |  | |  |  |
| **Education (N%)** |  | |  | |  |  | 0.12 | |  |  |
| Less than High School | 1(7) | | 0(0) | | 2(50) | 0(0) |  | |  |  |
| High School Degree or some college | 3(20) | | 4(40) | | 1(25) | 0(0) |  | |  |  |
| Any College Degree | 11(73) | | 6(60) | | 1(25) | 1(100) |  | |  |  |
|  |  | |  | |  |  |  | |  |  |
|  |  | |  | |  |  |  | |  |  |
|  |  | |  | |  |  |  | |  |  |
|  |  | |  | |  |  |  | |  |  |
|  |  | |  | |  |  |  | |  |  |
|  |  | |  | |  |  |  | |  |  |

^A^Significance determined via chi-square tests for categorical variables and t-tests for continuous variables

***Supplementary Table 2: Baseline Characteristics of Pediatric Replication Sample (N = 114)***

| Parental ACE Categories |  | No ACE ACE 1-3 | | ACE 4-6 | | ACE > 6 | | P-Value^A^ | | |
| --- | --- | --- | --- | --- | --- | --- | --- | --- | --- | --- |
|  | 41 | | 58 | | 13 | 2 |  | |  |  |
| **Age at Sample Collection** |  | |  | |  |  |  | |  |  |
| Age(SD) | 2.4(1.9) | | 2.6(2.1) | | 3.8(2.8) | 5.1(3.0) | 0.11 | |  |  |
| **Sex N(%)** |  | |  | |  |  |  | |  |  |
| Female | 15(38) | | 20(35) | | 5(38) | 0(0) |  | |  |  |
| **Parental Smoking Status N(%)** |  | |  | |  |  |  | |  |  |
| Ever Smoke? | 2(4.7) | | 2(3.4) | | 2(15) | 0(0) | 0.39 | |  |  |
| **Hispanic Ethnicity N(%)** |  | |  | |  |  |  | |  |  |
| Yes | 10(26) | | 16(27) | | 4(31) | 0(0) | 0.87 | |  |  |
| **Parental Depression N(%)** |  | |  | |  |  |  | |  |  |
| Ever diagnosed by a doctor? | 6(14) | | 12(22) | | 5(38) | 1(0.5) | 0.19 | |  |  |
| **Parental Anxiety N(%)** |  | |  | |  |  |  | |  |  |
| Ever diagnosed by a doctor? | 9(21) | | 15(27) | | 4(31) | 0(0) | 0.28 | |  |  |
| **Collection Method N(%)** |  | |  | |  |  |  | |  |  |
| Toothbrush | 8(19) | | 10(17) | | 1(8) | 0(0) | 0.28 | |  |  |
| Swab | 33(81) | | 47(83) | | 12(92) | 2(100) |  | |  |  |
|  |  | |  | |  |  |  | |  |  |
|  |  | |  | |  |  |  | |  |  |
|  |  | |  | |  |  |  | |  |  |
|  |  | |  | |  |  |  | |  |  |
|  |  | |  | |  |  |  | |  |  |
|  |  | |  | |  |  |  | |  |  |

^A^Significance determined via chi-square tests for categorical variables and t-tests for continuous variables

***Supplementary Table 3: Nominally Significant Correlation with RNA-sequencing Dataset Sample***

| **Gene** | **Pearson Correlation** | **P-Value** |
| --- | --- | --- |
| Maternal ACE_1-3_ | | |
| *NMNAT2* | 0.37 | 2.5e-2 |
| *HDAC4* | -0.33 | 4.7e-2 |
| *GAK* | -0.38 | 2.2e-2 |
| *MFAP3* | 0.35 | 3.5e-2 |
| *VARS2* | -0.64 | 3.5e-5 |
| *MAFK* | -0.41 | 1.4e-2 |
| *PON1* | 0.37 | 2.9e-2 |
| *TUBGCP5* | -0.49 | 2.5e-3 |
| *CLCN7* | 0.34 | 4.3e-2 |
| *GPR108* | -0.40 | 1.7e-2 |
| *TXNRD2* | 0.42 | 1.0e-2 |

***Supplementary Figure 5: Boxplots of CpGs within Replicated DMRs for discovery and replication cohort***

***
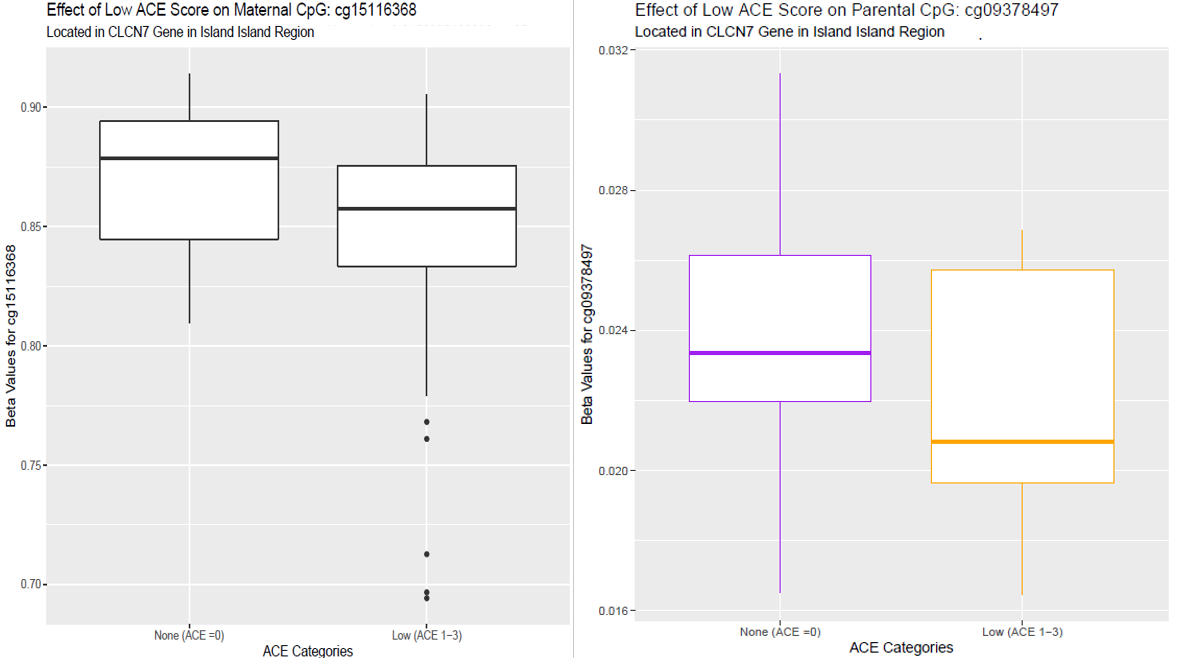
***

*Boxplots of beta values for CpGs significant in the CLCN7 DMR for MADRES and CHS*

***Supplementary Figure 6: Comparing the Marginal Effect in the Discovery and Replication Population***

**
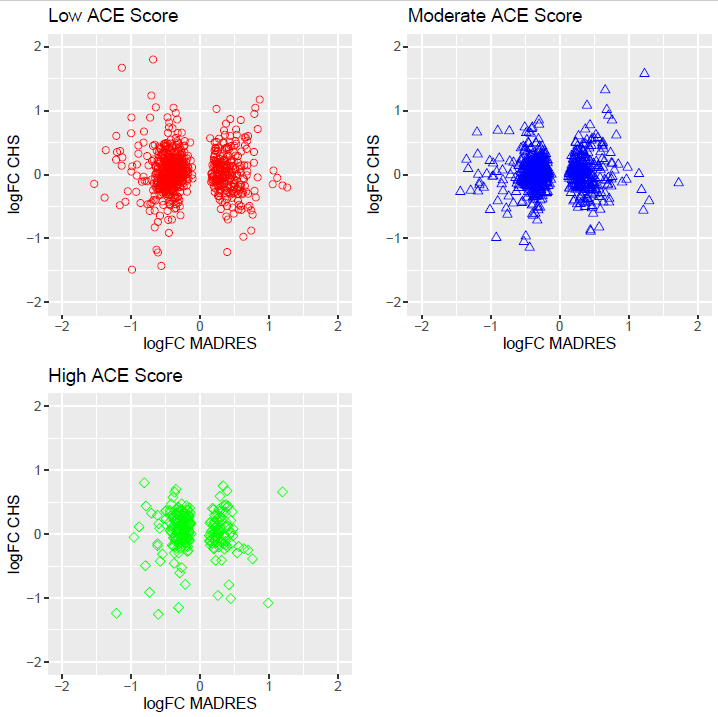
**

*Overlap in direction of log-fold change coefficients between significant MADRES probes and CHS across ACE categories.*

**Supplemental Text**

*Adverse Childhood Experiences (ACEs) Data Collection*

Distribution of ACEs in this study population may differ from those in the general population because the study sample was drawn from the MADRES pregnancy cohort and exclusion criteria include factors that themselves might have been affected by ACEs. The target population in MADRES was Hispanic individuals (defined as individuals with heritage from a Spanish-speaking country) with a uterus and was conditional upon recruitment from four prenatal care clinics in Los Angeles County, California between 2015 and 2018. Exclusion criteria included individuals who were HIV-positive, currently incarcerated, or more than 30 weeks gestational age at study entry. Given that ACEs may increase your risk of HIV infection, incarceration, miscarriage, and death, this could introduce a selection bias in the analytic population since individuals with higher ACEs were more likely to have been excluded from the analysis [3,4]. Such a selection bias could affect study findings as individuals most affected by ACEs are not included in the analysis, effectively reducing the magnitude of the effect estimate. Among individuals who had available DNA methylation data at two timepoints, 7% were missing ACE questionnaire data. Given the nature of the ACEs questionnaire, missing data may reflect resistance to reporting that may also be associated with higher ACE scores. Additionally, retrospective reporting is prone to misclassification and can be a result of random forgetfulness, the suppression of early life adversity memories, or the ability to recognize that one has experienced such adversity [5]. We expect retrospective non-differential misclassification to bias results towards the null, as individuals would be underreporting their true ACE score.

**Figure C1: Directed Acyclic Graph**

**
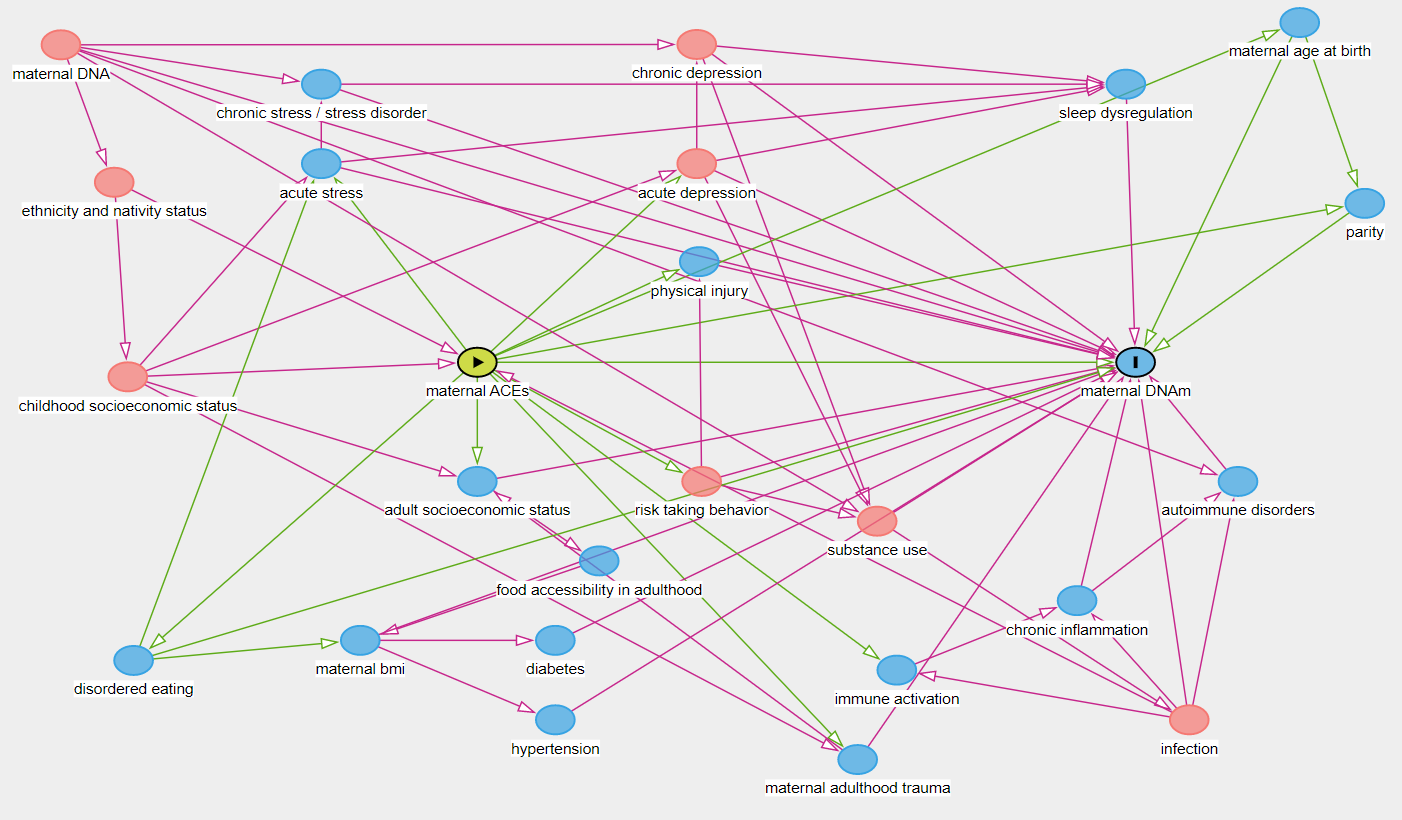
**

*Maternal ACEs as the green exposure node, and maternal DNAm as outcome. Causal paths are in green, and biasing paths are in red [13].*

There are several downstream pathways associated with early life adversity (ELA) notated on the directed acyclic graph (DAG) in Figure C1. For example, one pathway is causing risk taking behaviors, which in turn increases risk of substance use and subsequently addiction. Each of these nodes may affect maternal DNAm independently or have a cumulative pathway effect. Effects of addiction acting on maternal DNAm were adjusted for persistent cigarette smoking use in pregnancy. Nicotine use or cigarette smoking addiction is highly correlated with other types of substance use [6]. Mixture effects of multiple substances together may exert differential effects than the independent effects of these substances [7]. Intravenous drug use may increase risk of infection and chronic inflammation as well. Variability in maternal DNAm contributed by lifetime substance use may likely be not sufficiently adjusted for. Overall, effects from this pathway may suffer from residual confounding in a direction that would bias away from the null. This could also be contributed to by an underlying reporting bias, given the stigmatization of smoking consistently in pregnancy.

Another pathway is associated with disordered eating, which may result in fluctuation in body mass index (BMI), thereby affecting DNA methylation. BMI may also increase risk of diabetes and hypertension, also affecting DNA methylation profiles [8]. Disordered eating can also increase risk of stress, and therefore a chronic stress disorder, which also can affect DNAm [9]. Chronic stress disorder was not adjusted for in our population, given that only two individuals in the low ACE group displayed evidence for high perceived stress using the perceived stress scale (PSS) at the early trimester visit [10]. A sensitivity analysis was conducted for the maternal effect, and the *COMT* effect was robust to this analysis. Another path is associated with the effects of acute depression causing chronic depression–which may increase risk of sleep dysregulation–which we accounted for by adjusting for chronic depression. Finally, paths of ELA increasing risk of immune activation and chronic inflammation were not blocked or adjusted for. Therefore, our results reflect a cumulative path effect of immune activation and chronic inflammation. Importantly, ELA may increase your risk of infection (HIV, HPV, HSV, hepatitis), which may also increase risk of immune activation and inflammatory pathways [11]. Though HIV negative status was required for entry into the cohort, other more prevalent infections may be biasing our association. The effect of ELA on adulthood trauma is also unaccounted for in our analysis, which has the potential to bias our result in multiple directions. Additional confounders were also identified that did not act through ACE paths, including maternal age at birth, parity, ethnicity and nativity status, and genetics Using the DAG to inform our covariate adjustment, we have attempted to create an exchangeable study population based on the following confounding pathways to identify the effect of early life adversity on internalizing and externalizing behaviors affecting DNA methylation: adult socioeconomic status, maternal BMI, chronic depression, diabetes, hypertension, maternal DNA, maternal age at birth, parity, and ethnicity and nativity status.

**Mediation Analysis**

In addition to the assumptions of causal inference methods of exchangeability, positivity, and consistency, causal mediation also requires that there is no exposure-induced mediator-outcome confounding. This might be violated in our study sample by three pathways: maternal BMI, adult socioeconomic status, and adulthood trauma. To address this, we tested for any evidence of a differential overall indirect effect using a multiple mediators’ approach [12]. Briefly, the method fits two mediators and one outcome model. The outcome model considers the joint associations between both mediators and the outcome. This approach identifies the effect gained from pre-pregnancy BMI when considering it together with maternal *COMT.* This contrasts with considering the two mediators independently, where a separate outcome model would be fit for each mediator. The sensitivity analysis did not provide substantial evidence for maternal pre-pregnancy BMI acting as an exposure-induced mediator outcome confounder. To address potential exposure-induced mediator outcome confounding by adulthood socioeconomic status, a crude unadjusted mediation analysis was conducted restricted to pairs (N = 22) with the most prevalent adult income status in our population; results reflected the main effect with a confidence interval that did not overlap the null (95% CI = (0.0005, 0.6308)).

Figure C2.

**
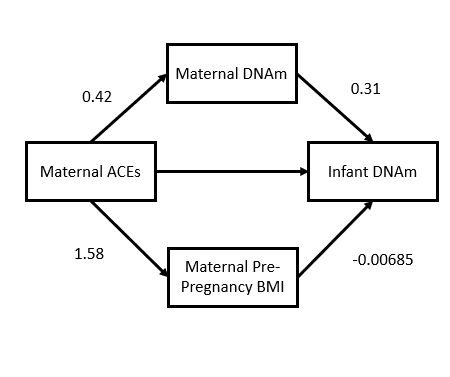
**

*Our results indicate an overall indirect effect for the joint contribution of ACEs acting on DNAm and pre-pregnancy BMI on neonatal DNAm to be 0.13 with a 95% confidence interval of (-0.0237,0.957).  This null result means there is no additional contribution of pre-pregnancy BMI on neonatal DNAm beyond what is contributed by maternal DNAm [12]. Overall, these indirect effects were consistent with our conclusions from our main effect result*

**Discussion**

We have attempted to satisfy the assumptions of exchangeability using a systematic literature review of the effects of ACEs on DNA methylation as a guide for our DAG development and selection of confounding pathways for control. We also added nodes to this DAG based on effects reported in the childhood maltreatment literature. Exchangeability, however, may be violated as a function of inadequate control for blocked paths as described above. Positivity was not violated as every individual has a childhood with a capacity for experiencing adversity. The assumption of consistency was violated, as all studies using childhood maltreatment in the context of causal effects are subject to the effects of time and circumstance of the specific childhood maltreatment experience(s) that cannot be adequately modeled.

**References**

1. Aryee MJ, Jaffe AE, Corrada-Bravo H, Ladd-Acosta C, Feinberg AP, Hansen KD, et al. (2014). “Minfi: A flexible and comprehensive Bioconductor package for the analysis of Infinium DNA Methylation microarrays.” Bioinformatics, **30**(10), 1363–1369. doi:10.1093/bioinformatics/btu049.
2. Zheng SC, Breeze CE, Beck S, Teschendorff AE. Identification of differentially methylated cell types in epigenome-wide association studies. Nat Methods. 2018 Dec;15(12):1059-1066. doi: 10.1038/s41592-018-0213-x. Epub 2018 Nov 30. PMID: 30504870; PMCID: PMC6277016.
3. Felitti VJ, Anda RF, Nordenberg D, Williamson DF, Spitz AM, Edwards V, et al. Relationship of childhood abuse and household dysfunction to many of the leading causes of death in adults. The Adverse Childhood Experiences (ACE) Study. Am J Prev Med. 1998 May;14(4):245-58. doi: 10.1016/s0749-3797(98)00017-8. PMID: 9635069.
4. Bastain TM, Chavez T, Habre R, Girguis MS, Grubbs B, Toledo-Corral C, et al. Study Design, Protocol and Profile of the Maternal And Developmental Risks from Environmental and Social Stressors (MADRES) Pregnancy Cohort: a Prospective Cohort Study in Predominantly Low-Income Hispanic Women in Urban Los Angeles. BMC Pregnancy Childbirth. 2019 May 30;19(1):189. doi: 10.1186/s12884-019-2330-7. PMID: 31146718; PMCID: PMC6543670.
5. Reuben A, Moffitt TE, Caspi A, Belsky DW, Harrington H, Schroeder F, et al. Lest we forget: comparing retrospective and prospective assessments of adverse childhood experiences in the prediction of adult health. J Child Psychol Psychiatry. 2016 Oct;57(10):1103-12. doi: 10.1111/jcpp.12621. PMID: 27647050; PMCID: PMC5234278.
6. Torabi MR, Bailey WJ, Majd-Jabbari M. Cigarette smoking as a predictor of alcohol and other drug use by children and adolescents: evidence of the "gateway drug effect". J Sch Health. 1993 Sep;63(7):302-6. doi: 10.1111/j.1746-1561.1993.tb06150.x. PMID: 8246462.
7. Nielsen DA, Utrankar A, Reyes JA, Simons DD, Kosten TR. Epigenetics of drug abuse: predisposition or response. Pharmacogenomics. 2012 Jul;13(10):1149-60. doi: 10.2217/pgs.12.94. PMID: 22909205; PMCID: PMC3463407.
8. Khaodhiar L, McCowen KC, Blackburn GL. Obesity and its comorbid conditions. Clin Cornerstone. 1999;2(3):17-31. doi: 10.1016/s1098-3597(99)90002-9. PMID: 10696282.
9. Yau YH, Potenza MN. Stress and eating behaviors. Minerva Endocrinol. 2013 Sep;38(3):255-67. PMID: 24126546; PMCID: PMC4214609.
10. Baik SH, Fox RS, Mills SD, Roesch SC, Sadler GR, Klonoff EA, et al. Reliability and validity of the Perceived Stress Scale-10 in Hispanic Americans with English or Spanish language preference. J Health Psychol. 2019 Apr;24(5):628-639. doi: 10.1177/1359105316684938. Epub 2017 Jan 5. PMID: 28810432; PMCID: PMC6261792.
11. Elwenspoek MMC, Kuehn A, Muller CP, Turner JD. The effects of early life adversity on the immune system. Psychoneuroendocrinology. 2017 Aug;82:140-154. doi: 10.1016/j.psyneuen.2017.05.012. Epub 2017 May 17. PMID: 28549270.
12. VanderWeele, Tyler and Vansteelandt, Stijn. “Mediation Analysis with Multiple Mediator” *Epidemiologic Methods*, vol.2,no.1,2014,pp.95-115. https://doi.org/10.1515/em-2012-0010
13. Textor J, van der Zander B, Gilthorpe MS, Liśkiewicz M, Ellison GT (2016). “Robust causal inference using directed acyclic graphs: the R package 'dagitty'.” International Journal of Epidemiology, **45**(6), 1887–1894. doi:10.1093/ije/dyw341.
